# Equity, Diversity and Inclusion (EDI) in Organ Transplantation: An ESOT Survey About EDI Within ESOT as an Organization and its Educational Activities, and Transplantation Research and Science

**DOI:** 10.3389/ti.2023.11331

**Published:** 2023-08-23

**Authors:** L. H. M. Pengel, M. Kaisar, S. Benjamens, M. Ibrahim, V. Ricci, M. I. Bellini, A. C. Breithaupt-Faloppa, C. Falk, H. Maple, L. Marson, F. Ortiz, V. Papalois, D. Paredes, A. Forsberg

**Affiliations:** ^1^ Erasmus MC Transplant Institute, University Medical Center, Rotterdam, Netherlands; ^2^ Peter Morris Centre for Evidence in Transplantation, Nuffield Department of Surgical Sciences, University of Oxford, Oxford, United Kingdom; ^3^ Nuffield Department of Surgical Sciences, University of Oxford, Oxford, United Kingdom; ^4^ Department of Surgery, Ikazia Hospital, Rotterdam, Netherlands; ^5^ Manchester Royal Infirmary, Manchester, United Kingdom; ^6^ European Society for Organ Transplantation, Padua, Italy; ^7^ Department of Surgery, Sapienza University of Rome, Rome, Italy; ^8^ LIM-11, Hospital das Clinicas da Faculdade de Medicina da Universidade de Sao Paulo, Sao Paulo, Brazil; ^9^ Institut für Transplantationsimmunologie, Medizinische Hochschule Hannover, Hannover, Germany; ^10^ Guy’s and St Thomas’ NHS Foundation Trust, London, United Kingdom; ^11^ The Edinburgh Transplant Centre, Royal Infirmary of Edinburgh, Edinburgh, United Kingdom; ^12^ Abdominal Center Unit, Nephrology, Helsinki University Hospital, Helsinki, Finland; ^13^ Imperial College Renal and Transplant Centre, Hammersmith Hospital, Imperial College Healthcare NHS Trust, London, United Kingdom; ^14^ Donation and Transplant Coordination Unit, Hospital Clinic Barcelona, University of Barcelona, Barcelona, Spain; ^15^ Department of Health Sciences, Lund University, Lund, Sweden; ^16^ Department of Thoracic Surgery, Skane University Hospital, Lund, Sweden

**Keywords:** diversity and inclusion, equity, solid organ transplant, survey, transplantation professionals

## Abstract

The European Society of Organ Transplantation (ESOT) strives to promote equity, diversity, and inclusion (EDI) across all its activities. We surveyed the transplant community’s experiences and perspectives regarding EDI within ESOT as an organization and its educational activities, and research in general. A total of 299 respondents completed the questionnaire. About half agreed that ESOT’s Executive Committee, Council, and Sections/Committees are diverse and inclusive (51%) and that ESOT promotes EDI in its live and digital educational activities (54%). Forty percent of respondents agreed that scientific and clinical trials in the field of transplantation are diverse and inclusive. Despite the wide distribution of the survey, most of the respondents self-identified as White and were either physician or surgeon. However, the results contribute a unique insight into the experiences and perspectives of the transplantation community regarding EDI. Whilst ESOT is committed to the principles of EDI, perceptions and the high number of proposals show the apparent need to prioritize efforts to embed EDI across ESOT and transplantation science. These data should constitute a starting point for change and provide guidance for future efforts to promote EDI within the transplantation community.

## Graphical Abstract

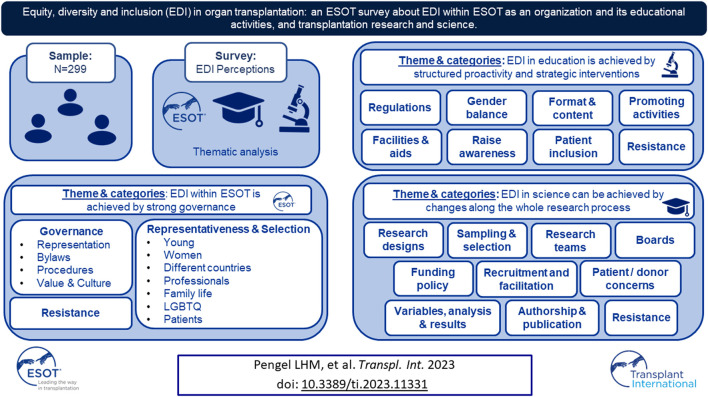

## Introduction

Societal evolution has increased awareness of the challenges involved in achieving equity, diversity, and inclusion (EDI) in professional environments. Accordingly, many medical organizations have acknowledged their role in promoting and improving EDI to develop an inclusive workforce. Studies on EDI within clinical and academic transplantation have revealed alarming results. A survey conducted by the International Liver Transplant Society showed a low rate of female leadership (8.2%), a high rate of professionals who experienced racial and/or gender-related discrimination (34.7%), and a low rate of support after discrimination for the victims (43.7%) [1]. A retrospective analysis of transplant centers in the United States showed that only 8.5% of transplant surgical directors were female, 5% were Black and a majority were non-Hispanic White (55%) [2]. Gender disparity was also evident in scientific and clinical transplant research. Studies have shown a higher percentage of male first and last authorships (63.8% and 69.8%, respectively) in high-impact scientific publications, a higher percentage of male versus female editors in chief (82% and 18%, respectively), less female author citations, and less external funding awards to female researchers [3, 4].

A review of racial disparities in the US transplant surgery workforce showed minimal improvement from 2000 to 2013 [5]. The number of Black transplant surgeons increased from 2% to 5.5%, while the White to Non-White transplant workforce ratio increased by 35%. In the US heart transplant workforce, a small increase in the percentage of Non-White surgeons was seen between 2000 and 2020, where the percentage of Black surgeons changed from 0.7% to 2.0%, Hispanic surgeons from 2.3% to 4.4%, and Asian surgeons from 8.2% to 22.8% [6]. Failure to adhere to the principles of EDI harms scientists, trainees and patients, and leads to unequal access to leadership, career advancement opportunities and compensation [7, 8].

These data demonstrate that awareness and promotion of EDI within the field of organ transplantation are urgently needed. We therefore explored the EDI perceptions of the European transplant community to identify areas for improvement in promoting awareness of EDI.

## Methods

The European Society for Organ Transplantation (ESOT) Diversity & Inclusion Advisory Group was formed to advance diversity and inclusion within ESOT. The Group consisted of ten members with various backgrounds and roles within ESOT from eight different countries both within and outside Europe. A questionnaire was designed to survey perceptions regarding the strategies required to promote EDI within ESOT. For the questionnaire EDI was broadly defined as concerning gender, sexual orientation, ethnic and racial background, immigration status, ability/disability, socio-economic status, and the patient perspective.

The questionnaire collected demographics and EDI perceptions regarding several topics (Supplementary Table S1). This paper presents the data on the following topics: 1) ESOT’s promotion of EDI within the Executive Committee, Council, and Sections/Committees; 2) ESOT’s promotion of EDI in its live and digital educational activities, scientific content, and attendance; and 3) EDI in clinical research and science. Perceptions were scored on a 5-point Likert Scale ranging from “strongly agree” to “strongly disagree.” Open-ended questions collected up to three proposals for promoting EDI.

The online questionnaire was open from 5 May to 30 June 2021 and again from 1 October to 1 December 2021 to solicit further responses. The questionnaire was distributed through ESOT newsletters and various social media channels.

Data were analyzed according to qualitative research methods. Open-ended answers were analyzed by AF who conducted content analysis of the explicit written words and their meanings, symbolic qualities, and expressive content. In line with Krippendorff, the content analysis summarized surface features of the meaning units and interpreted the content [9]. Thus, the underlying meaning in each passage was illustrated by themes, while the manifest data were organized into categories and subcategories. First, every statement was read in detail, which provided a general sense of the content. Thereafter, the responses were divided into meaning units, i.e., single words, parts of, and whole sentences, and then condensed. The content of the condensed meaning units was formulated into categories and sub-categories. In line with qualitative research methods, numerical data were not presented for these categories and sub-categories. The interpretations were based on a holistic analysis of the content in each category and included several themes. The themes can be seen as a thread of meaning that recurs in the content of the categories.

## Results

Two hundred and ninety-nine respondents completed the questionnaire. Most respondents were aged 35–44 years (36.2%), self-identified as White (83.5%), physician (27.4%), and described themselves as female (55.5%). Respondents came from 38 countries with the United Kingdom (33.9%), Spain (14.4%), Italy (8.7%), France (5.7%), Netherlands (5.0%), and Switzerland (3.4%) being the most frequently reporting countries (Table 1).

**TABLE 1:** Demographics of the survey respondents (*n* = 299).

Characteristics	Responses (%)
Age	
18–24 years	5 (1.7%)
25–34 years	47 (15.8%)
35–44 years	108 (36.2%)
45–54 years	83 (27.9%)
55–64 years	48 (16.1%)
65+ years	7 (2.4%)
Missing	1 (0.3%)
Gender	
Female	166 (55.5%)
Male	130 (43.5)
Non-binary	2 (0.7%)
Prefer not to say	1 (0.3%)
Country (≥3 responses)	
United Kingdom	101 (33.9%)
Spain	43 (14.4%)
Italy	26 (8.7%)
France	17 (5.7%)
Netherlands	15 (5.0%)
Switzerland	10 (3.4%)
Belgium	9 (3.0%)
Turkey	7 (2.4%)
United States of America	6 (2.0%)
Australia	5 (1.7%)
Austria	5 (1.7%)
Finland	5 (1.7%)
Mexico	5 (1.7%)
Brazil	4 (1.3%)
Germany	4 (1.3%)
Sweden	4 (1.3%)
Hungary	3 (1.0%)
India	3 (1.0%)
Russian Federation	3 (1.0%)
Ethnic background	
White	248 (83.5%)
Asian: Indian	10 (3.4%)
Mixed: White and Black African	3 (1.0%)
Mixed: White and Black Caribbean	2 (0.7%)
Mixed: White and Asian	2 (0.7%)
Arab	6 (2.0%)
Asian: Pakistani	6 (2.0%)
Black/African/Caribbean	5 (1.7%)
Asian: Chinese	1 (0.3%)
Other / prefer to self-describe	12 (4.0%)
Prefer not to say	2 (0.7%)
Missing	2 (0.7%)
Background	
Physician	82 (27.4%)
Surgeon	71 (23.7%)
Nurse including specialist nurse	44 (14.7%)
Scientist	27 (9.0%)
Transplant coordinator	16 (5.4%)
Patient	15 (5%)
Allied health care professional	14 (4.7%)
Pharmacist	3 (1.0%)
Patient advocate	2 (0.7%)
Ethics	2 (0.7%)
Caregiver	1 (0.3%)
Living donor	1 (0.3%)
Other	21 (7.0%)

### EDI in the Executive Committee, Council, and Sections and Committees

**FIGURE 1:**
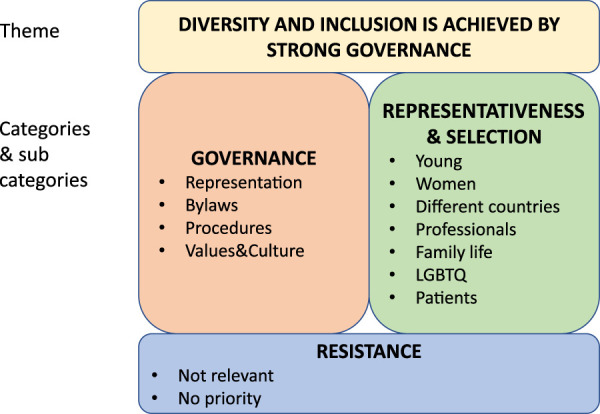
The respondents’ perspectives on how the European Society for Organ Transplantation (ESOT) can promote diversity and inclusion in the executive committee, council, and sections and committees, labelled according to overall theme, categories and subcategories.

Fifty-one percent of respondents either agreed or strongly agreed that ESOT is a diverse and inclusive organisation regarding its Executive Committee, Council, and Sections/Committees, 33% neither agreed or disagreed, and 15% either disagreed or strongly disagreed. The main theme among the proposed ideas was that EDI is achieved by strong governance, which consisted of the subthemes “Governance” and “Representativeness and Selection.” “Governance” included, for example, bylaws and procedures and “Representativeness and Selection” young professionals, women and patients (Figure 1).

#### Governance

The category “Governance” included perceptions and proposals regarding representation, where it was perceived as important to outline structures in an organic, open, and transparent way when recruiting Executive Committee members. It was suggested that senior management should always include at least one woman. Balanced gender representation was strongly emphasized and preferred in any selection process and may be achieved by quota, leadership training, and by giving extra points to women and/or ethnic minorities. Furthermore, observer positions on committees for trainees were welcomed. Governance also includes bylaws in which EDI regulations are outlined. Respondents stated that diversity should be visible in all layers of the organization and bylaws needed to reflect diversity goals for the whole organization, including Sections and Committees.

To support EDI, valid procedures are required. Awareness can be increased by monitoring EDI across Sections and Committees and making the data public. Other suggestions included increasing transparency in selection procedures, being mindful when evaluating candidates, anonymizing applications, and reaching out to persons outside of ESOT. It was felt that ESOT also needs to encourage and support applications from less established professionals. Using different languages in meetings, offering implicit bias training, limiting the tenure of Council members, and promoting a bottom-up instead of a top-down organization were also suggested.

Lastly, governance is about promoting values and building an inclusive culture. This can be done by ensuring diversity in award winners and highlighting diversity as a value of the organization. Young professionals should be nurtured by means of programs and ESOT should highlight initiatives for implementation of these programs, and for recruiting and developing talented professionals of all genders. Finally, the establishment of an EDI and outreach committee may ensure that EDI is always on the agenda.

#### Representativeness and Selection

Several groups were highlighted as underrepresented or as minority groups within the ESOT community, which participants felt should be targets for selection and outreach activities. Young professionals are regarded as persons in need of support and promotion, and who should participate in working groups alongside more experienced professionals. The importance of approaching young professionals from outside the US and Europe. especially from lower income countries, was also stressed. A key message was that women in general, and non-White women in particular, should be encouraged to participate and apply for awards to achieve a 50/50 male-female ratio.

Recruitment of professionals from different countries, backgrounds, and ethnicities was also highlighted as an important area for improvement. Ethnic diversity means including professionals from Black, Asian, Minority Ethnic (BAME), Latin American and non-European backgrounds. Suggestions included grants which could be offered to non-European researchers to stimulate inclusion. One way to prevent bias would be to use blinded selection procedures and elections, which would simultaneously facilitate inclusion of professionals with disabilities. Improvements in diversity may be achieved by actively encouraging people of all backgrounds and genders to apply for positions within ESOT.

Professionals with families and young children need to be supported by initiatives that facilitate combining career and family life such as access to digital learning solutions, e.g., webinars and online courses. Furthermore, increased lesbian, gay, bisexual, transgender, queer and intersex (LGBTQI+) visibility and representation were requested to show ESOT’s support of this group.

There is also a need for patient inclusion, in particular independent patients free of policy agendas. Accepting patients in the Council and Sections and Committees would enable them to contribute to decision-making processes. Finally, some respondents opposed the efforts to ensure EDI, arguing that EDI should not be promoted at all.

### EDI in ESOT’s Live and Digital Educational Activities

**FIGURE 2:**
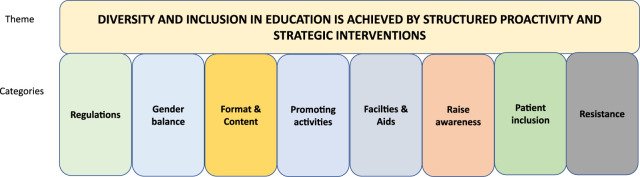
The respondents’ perspectives on how the European Society for Organ Transplantation (ESOT) can promote diversity and inclusion in its live and digital educational activities regarding/in terms of both the scientific program and attendance, labelled according to overall theme and categories.

Fifty-four percent of respondents either agreed or strongly agreed that ESOT promotes diversity and inclusion in its live and digital educational activities, 36% neither agreed or disagreed, and 10% either disagreed or strongly disagreed. The main theme among the proposed ideas was that EDI is achieved by structured proactivity and strategic interventions. Eight subthemes were identified which included “Regulations,” “Gender balance,” “Format and content,” “Promoting activities,” “Facilities and aids,” “Raise awareness,” “Patient inclusion” and “Resistance” (Figure 2).

#### Regulations

Respondents felt that ESOT needed to develop policies and critically review whether the inclusion policies were truly representative. An EDI position statement might be useful as metrics for all types of committees.

#### Gender Balance

There was a strong need for gender balance and ESOT should be encouraged to consider gender parity when setting up mixed working groups. Gender balance should be aimed for in all panels, session chairs, and should be ensured throughout the scientific programme.

#### Format and Content

There are many suggestions for improving both the format and content of educational activities. These include more webinars, digital courses, online support for learning, and making use of, e.g., Twitter, which is easily accessible via smartphone. Respondents also requested specific EDI educational activities. Educational videos on YouTube and face-to-face online sessions were also suggested.

Inclusion could be promoted by organizing congress hubs in Eastern Europe and emerging countries, which could be combined with lectures from different parts of Europe. Educational activities should be free of scientific jargon so that all those involved, e.g., patients, could understand the content, which may facilitate broader participation.

Diverse panels led by underrepresented members could be included at scientific meetings to generate discussion groups. Education on EDI is needed and ESOT should ensure that the scientific program reflects the diversity of the society membership. There could also be focused sessions on EDI at the ESOT congress to share good practice. Clear guidance on gender and ethnic participation in the scientific programs should be available during the planning and the topic of ethnic and social differences could be addressed at congresses.

#### Promote Activities

Effective promotion of activities would facilitate attendance during various educational activities and the ESOT congress. The respondents highlighted the fact that participants have different backgrounds, which may not be the typical ones seen in transplantation medicine. There could also be a targeted quota for attendance or organizing committee membership of non-EU members. Low entrance fees for underrepresented groups, e.g., nurses, may stimulate their participation. More diverse moderators and speakers from different countries, avoidance of inviting the usual speakers and no events without female moderators and speakers were also requested. Respondents stress the importance of balancing the age, gender, and backgrounds of speakers while preserving the scientific value of the activity. This requires a conscious strategy regarding who to invite, e.g., speakers from Eastern Europe, non-ESOT members or leaders of the different minority groups.

Several respondents emphasized the importance of low registration fees to facilitate participation from underdeveloped or non-EU countries, trainees, young professionals, nurses, and allied health professionals. Extending invitations to, e.g., countries in Africa and Asia, would increase diversity and lead to new perspectives. To nurture the next-generation, the importance of their role in educational activities was emphasized, as was their need for support, targeted interventions, membership of scientific program committees, and invitations to chair meetings. In this context, social media could be utilized for broader dissemination and communication.

#### Facilities and Aids

Specific facilities are needed for attendees with children. Provision of a quiet space should be mandatory at every meeting to allow breastfeeding or pumping, or for pregnant women to rest. Childcare facilities were also requested.

By using languages other than English at meetings the diversity of attendees would increase. To make the scientific program more accessible for people with limited health literacy, lay terms could be used in certain sessions and materials developed for audiences with specific needs.

#### Strategies to Raise Awareness and Increase Inclusion

The respondents provided ample suggestions for how to raise awareness of EDI. ESOT should address LGBTQI+ issues and organize a task force to promote their inclusion in the community. Respondents also commented that transplant nurses are not represented in ESOT educational activities or reflected in the membership, which could be improved by creating education for nurses.

Targeted grants might be useful for engaging societies from countries in Africa, Asia, and the Middle East. Diverse role models are needed, and these should be highlighted through personal interviews and emphasizing their achievements. Furthermore, ESOT should utilize patients with medical skills and experience as public speakers, which would benefit the outreach to patients.

#### Patient Inclusion

The call for patient participation is strong and respondents suggest that patients should be part of all ESOT activities. ESOT needs to reach out to different communities representing patient populations. Transplant care professionals and patients should interact for mutual learning and patients can assist with specific recommendations.

#### Resistance

Some respondents persistently argued that ESOT should stop wasting time with surveys about EDI. In their opinion it is not the job of ESOT to promote EDI.

### EDI in Science and Clinical Trials

**FIGURE 3:**
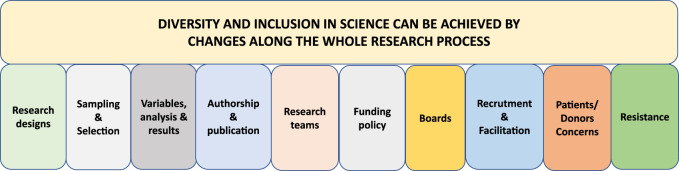
The respondents’ perspectives for change along the research process to achieve EDI in science and clinical trials, labelled according to overall theme and categories.

Forty percent of respondents either agreed or strongly agreed that science and clinical trials in the field of transplantation are diverse and inclusive, 28% neither agreed or disagreed, and 32% either disagreed or strongly disagreed. The main theme among the proposed ideas was that EDI can be achieved by changes along the whole research process. Ten subthemes were identified which included “Research designs,” “Sampling and selection,” “Variables, analysis and results,” “Authorship and publication,” “Research teams,” “Funding policy,” “Boards” and “Recruitment and facilitation,” “Patient and Donor concerns” and “Resistance” (Figure 3).

#### Research Designs

Respondents pointed out that studies pertaining to transplantation are prioritized over studies on donation and that more studies about inequalities are needed. Another area of research interest is pregnancy after transplantation, as reproductive health in relation to transplantation involves both gender and equity aspects.

Respondents suggested that each research project should describe how EDI will be implemented. Sex and gender balance should be addressed when designing trials as opposed to analyzed *post hoc*. ESOT could promote research in social and health sciences and encourage inclusion of baseline equity analytics in trial reports. Longitudinal surveys integrating all parameters would be welcomed as well as diversified trials that include all possible treatment groups. Common co-morbidities should be accepted as inclusion criteria and ESOT should also encourage qualitative research designs to complement traditional quantitative designs. Multicenter studies across continents will help to increase diversity in research. Finally, researchers are encouraged to consider niche studies for certain populations not represented in larger studies and diversity in case studies.

#### Sampling and Selection

Diversity starts at patient recruitment and could be directed at oversampling disadvantaged groups and gender balance. Animal research should balance sex in studies. Overall, there was a need to include marginalized populations and focus on those disadvantaged in society, e.g., Black and Asian ethnicity, and economically or educationally disadvantaged participants. Recruitment of minority staff would help to increase diversity among study populations. Incentives for patients show appreciation of their time and experience. Longitudinal designs should ensure that gender, race, and socio-economic status are reported baseline characteristics. Informants also raised that entirely European studies will always be partly biased.

#### Variables, Analysis and Results

Subgroup analyses by gender and race should be reported and the impact on BAME patients commented on. Possible biological diversity among women and various ethnic minorities needs to be considered. Finally, some low-income countries cannot afford certain treatments, which limits the worldwide application of results.

#### Authorship and Publication

Reviewer bias could be minimized by removing information that could identify the gender or ethnicity of authors. Respondents feel that access to publishing in high quality journals should be based on the quality of the manuscript, not on the country of origin. To promote publications from young researchers, journals may consider reduced publication fees. Respondents suggest developing appropriate peer review and publishing standards, e.g., justification of non-representative samples.

#### Research Teams

There were proposals about how to organize research teams and that promotion of women or minorities in clinical trial teams should be mandatory. A way to increase inclusion would be to create overseas committees as well as develop and practice EDI guidelines for forming research teams.

#### Funding Policy

There were many proposals to enable funding policies that promote EDI. One suggestion was to increase funding for projects originating outside of prestigious transplant centers and funding for principal investigators from underrepresented ESOT members. Another was to provide grants that address scientific questions related to EDI. In line with this, it was also suggested that grants should be made available for transplant surgeons and physicians from developing countries. It was specifically mentioned that both trainees and women would benefit from more funding options and that fellowships should be evenly distributed between male and female applicants. A sustainable funding framework could be developed by critically reviewing the funding allocation system and robustly assessing barriers to minority or underrepresented groups. Such a system should also provide grants to returning caregivers, scientists, and clinicians after a career break.

#### Boards

Research boards have the potential to influence the conduct of research and ensure adaptation of EDI principles in studies. It was stressed that research boards should not be all male or White panels and should include women to achieve representative steering committees. ESOT should organize a working group to focus on studies where EDI is the main subject and issue a public statement regarding their EDI policy. By offering bias training in the educational program, awareness would increase and established norms can be scrutinized and questioned. In general, it is considered important to create an inclusive research culture.

#### Staff Recruitment and Collaboration

The variety of proposals and comments on recruitment and collaboration was extensive and highlighted the importance of EDI awareness when recruiting staff. Online and offline interactions between ESOT and other society members from across the globe could facilitate collaboration, especially with non-continental and Eastern Europe, and exchange programs could also be established.

Opening ESOT membership to other specialties, e.g., anesthesia, was also suggested. Transplant surgeons and physicians from outside Europe and from underrepresented countries could be invited by ESOT to take part in scientific activities and clinical trials and be offered authorship. More scientific options for transplant coordinators, nurses, and allied health professionals (AHPs) are requested as they are underrepresented in scientific activities.

There were many options identified to enable research in real-world practice. Respondents suggested that ESOT may simplify access to resources, e.g., publications or journals, for young scientists and offer funding for scientists or researchers to become principal investigators. Information about ongoing or potential research projects could be shared via social media or other channels. Knowledge exchange is highly appreciated and gender equity leadership in research should be promoted. Meetings between researchers and clinicians promote exchange of ideas. A career path program would be helpful as well as facilitating staff mobility across Europe through a mentorship program that promotes EDI.

Respondents feel that sex and gender research should be regarded as high-profile and that ESOT should actively support and engage in clinical research that investigates gender, BAME or other diverse characteristics. The respondents suggest that professionals from low-income countries should participate in editorial groups to facilitate real-world clinical guidelines and trials.

ESOT could organize an EDI hub focusing on how to improve questionnaires studying this topic in relation to transplantation, donation, and different minorities, as well as considering the impact of religion on transplantation. EDI research could be a mandatory session at scientific meetings.

#### Patient/Donor (Living and Deceased) Concerns

Understanding issues from patient perspectives and embracing the patients’ point of view or concerns, e.g., whether strong medications are right for them, were considered important. A diverse pool of patients to support clinical trials could be created and patients should be involved from the early stages of trial design. There is a need for wider representation of both donors and recipients in trials, e.g., both the donating and non-donating families should be represented, as well as trials on the impact of donor-recipient mismatch. Finally, it was highlighted that research should aim for quality not quantity in organ allocation and improve outcomes in kidney transplantation by giving this solid organ transplantation life-saver status. Absence of this status prevents the initiation of new drug trials, often immunosuppression, and supports the idea of dialysis as a back-up, to the detriment of the patient.

#### Resistance

There were perceptions that promoting EDI is not at all essential for the goals of ESOT. The commitment should be to science and not to promoting EDI. There were also suggestions of racism against White people as well as sexism against men. Focusing on EDI was considered completely unnecessary.

## Discussion

The survey presented in this article attracted many responses and suggestions. Around half of respondents feel that ESOT promotes diversity. Nonetheless, half of the respondents do not agree that ESOT promotes diversity. The data demonstrate an EDI imbalance within ESOT and within transplantation science and clinical trials in general, and it appears clear that a significant proportion of respondents are dissatisfied with the current culture and lack of initiatives to increase EDI.

Despite this situation, EDI is not at the centre of the conversation within ESOT. Thus, we should reflect on the reasons for this. The ESOT culture is characterized by a strong drive for innovation and by a professional, collegial, and friendly climate. The latter could be described as a culture of “niceness,” a feature which, understood in conventional terms, is obviously very important for the functioning of a social group. At the same time, however, if the wish to be nice (or the expectation that people should be nice) makes it difficult for some people to speak up when there is a problem, then “niceness” can have problematic implications.

According to Sommers [9], niceness is our “most fundamental social dysfunction”; in organizations with high ambitions, it can be toxic and disabling insofar as the imperative to be nice can potentially silence those who are not in power and thus maintain the *status quo*. Furthermore, niceness is powerfully reinforced by spoken and unspoken discourses that control who can speak and when, and whether this voice will be heard and responded to. Niceness could create barriers to honest and potentially uncomfortable conversations [10]—precisely the type of conversations needed to address the lack of EDI. Our data show that respondents feel that these conversations are needed to ensure that women, patients, professionals from BAME backgrounds, nurses and AHPs are equally represented within ESOT, in science and clinical trials. A small part of the results demonstrated the need to maintain the *status quo* through resistance to EDI and the overall purpose of this survey.

Questioning EDI imbalances is often viewed as disruptive, which in turn is considered the opposite of “being nice.” Encouraging others to “be nice” and not to “rock the boat,” however, can serve to ensure that people remain silent when they should speak out and to avoid addressing the issues they raise. By conducting this survey, a voice was potentially given to the whole transplantation community, which *per se* promoted inclusion and diversity. In order to embrace and benefit from EDI, people have to engage in uncomfortable conversations. This requires the development of skills in initiating and facilitating respectful discussions, and inclusive leadership to drive change [11].

In environments shaped by niceness, speaking out can involve considerable personal risk: challenging the *status quo* can be taken as breaching the code of niceness, causing the exclusion or marginalization of those who speak out. The survey responses were confidential; thus, no codes could be violated. However, the lack of representativeness among the respondents might reflect a culture in ESOT where underrepresented groups perceive a barrier to speaking up.

The literature suggests that cultures of niceness disproportionately affect people from minority groups. Perlow [12] argues that niceness is not harmless or benign and instead positions niceness as a racialized and gendered tool used to disguise power relationships and a powerful means to silence and oppress people of color. This should be evident to many healthcare professionals because, despite the mantras of EDI that are often prominent in the statements of professional healthcare organizations, we still see widespread inequity and lack of inclusion.

The findings of this survey are mainly in line with previous research [1, 5]. Thus, it might be fair to argue that we have a clear picture of the challenges related to EDI in the transplant community. Deliberate actions are warranted to address the *status quo*, including encouraging honest (and sometimes uncomfortable) conversations to promote a culture change within the Society; addressing the lack of diversity in the Society’s leadership at all levels, with particular attention to the Executive Committee; developing easy and accessible tools to maintain persistent awareness of EDI as well as to prevent unnecessary bias as presented in Table 2.

**TABLE 2:** Examples of tools to raising awareness about equality, diversity and inclusion when planning learning, scientific meetings or communication.

Five core questions when formulating cases for problem-based learning
1. What is the learning outcome from the case?
2. What happens if the person/patient in the case has another sex? For example, changing from a man to a woman
3. What happens if the person/patient in the case has another name? For example, changing from Steve to Ahmed
4. What happens if the person/patient in the case has another next of kin? For example, changing from a female spouse as a caregiver to a male spouse
5. What happens if the person/patient in the case has another profession? For example, changing from an office executive to a carpenter or baker
**Five core questions when planning a scientific meeting or a webinar**
1. What is the learning outcome from the meeting?
2. Is there a balanced gender distribution among possible presenters and moderators? The gender balance should be at least 60%/40%
3. What happens if the keynote speaker is a woman instead of a man?
4. What happens if the person presenting research or keynote speaker has a profession other than that of a physician or surgeon? For example, changing from a physician or surgeon to a nurse or an allied health professional
5. Would it be possible to include patients in the programme? If not, what would be the reason for this?
**Five core questions when planning communication**
1. What is the key message in the communication regarding equality, diversity, and inclusion?
2. What happens if the person on the screen or providing the message has a different ethnicity than White?
3. What happens if the person on the screen or providing the message has another sex? For example, changing from a man to a woman
4. What happens if the person on the screen or providing the message is physically disabled or overweight?
5. What happens if a queer person delivers the message?

This study has several limitations. In the context of EDI, it is problematic that most respondents identified themselves as White and that minority groups were underrepresented. We did not ask if respondents were ESOT members or whether they were familiar with ESOT as an organization. Respondents unfamiliar with ESOT may have opted to skip the question or respond neutrally (neither agreed or disagreed). Even so, EDI concerns were clearly outlined by the respondents. Despite the option of providing open-ended answers there was limited room for in-depth elaboration of perceptions or statements. Nevertheless, the data were rich and provided an extensive number of meaning units illustrating the engagement for EDI aspects in ESOT. Almost 300 professionals from 38 countries might also be viewed as a strength, providing a broad representation of healthcare cultures. To be true to the qualitative method we deliberately chose not to quantify the number of perceptions in each category to emphasize the quality of the content. One purpose of the survey was to increase awareness and create engagement and as such the aim can be considered achieved.

In conclusion, this survey provided ample suggestions on how to raise awareness about EDI in ESOT. The number of proposals on how to improve the current situation suggests a strong motivation in the transplant community to work in a context where EDI is consistently on the agenda. Since the survey was held in 2021, ESOT has made efforts to improve EDI, for example, by ensuring gender balance in the scientific program committee and faculty of the upcoming 2023 ESOT congress. ESOT’s vision statement includes aspirations to “promote scientific advancement,” “deliver career advancement opportunities to all healthcare professionals” and “promote equitable access to transplantation and related therapeutic strategies.” Therefore, we feel that ESOT has a moral obligation to not only adhere to the EDI principles within all levels and activities of its organization but to also take a leading role in creating awareness and drive further change regarding EDI. This change requires a collective change of beliefs, values and attitudes within the transplantation community.

## Data Availability

The raw data supporting the conclusion of this article will be made available by the authors, without undue reservation.
