# Supplementary material for: Equity, Diversity and Inclusion (EDI) in Organ Transplantation: An ESOT Survey About EDI Within ESOT as an Organization and its Educational Activities, and Transplantation Research and Science
Source: Transpl Int. 2023 Aug 23;36:11331. doi: 10.3389/ti.2023.11331 (PMC10481529; doi:10.3389/ti.2023.11331)
Supplement: Supplementary file 1 [file DataSheet1.docx]

Equality, diversity, and inclusion survey

ESOT is committed to promoting diversity and inclusion within the society and its activities, the transplantation community at large, clinical research, and patient care. This survey aims to collect your ideas regarding which strategies are required to facilitate, promote and action diversity and inclusion across all these areas.

The survey takes about 10 minutes to complete and all responses will be kept anonymous.

For the purpose of the survey we consider a broad definition of diversity and inclusion which includes but is not limited to the following key points of focus:

- Ability/disability
- Ethnic and racial background
- Gender
- Immigration status
- Patients and caregivers
- Sexual orientation
- Socio-economic status

We thank you in advance for your contribution.

Sincerely,

The ESOT Team

**DEMOGRAPHICS**

**1. Age:**

- - 18-24y
  - 25-34y
  - 35-44y
  - 45-54y
  - 55-64y
  - 65y +

**2. What is your gender?**

- Female
- Male
- Non-binary
- Prefer not to say
- Prefer to self-describe

**3. In what country do you live?**
[list of world countries]

**4. What is your ethnic background?**

- Arab
- Asian: Bangladeshi
- Asian: India
- Asian: Korean
- Asian: Pakistani
- Black/African/Caribbean
- Mixed: White and Black Caribbean
- Mixed: White and Black African
- Mixed: White and Asian
- White
- Prefer not to say

**5. Background**

- Allied health care professional
- Caregiver
- Nurse
- Patient
- Pharmacist
- Physician
- Scientist
- Surgeon
- Transplant coordinator
- Other (please specify)

**DIVERSITY AND INCLUSION IN ESOT**

**6. Regarding its Executive Committee, Council, and Sections and Committees*, ESOT is a diverse and inclusive organisation.**

*** ESOT’s Sections include ECTORS, ECTTA, EDTCO, EKITA, ELITA, ELPAT, EPITA, VCA and the Committees include the Education, Basic Science, ETAHP and YPT committees.**

- Strongly agree
- Agree
- Neither agree nor disagree
- Disagree
- Strongly disagree

**7. Please list up to three proposals how ESOT can promote diversity and inclusion in the Executive Committee, Council, and Sections and Committees.**

**8. ESOT promotes diversity and inclusion in its live and digital educational activities, regarding both the scientific programme and attendance.**

- Strongly agree
- Agree
- Neither agree nor disagree
- Disagree
- Strongly disagree

**9. Please list up to three proposals how ESOT can promote diversity and inclusion in its live and digital educational activities, regarding both the scientific programmes and attendance.**

**SCIENCE AND CLINICAL TRIALS**

**10. Science and clinical trials in the field of transplantation are diverse and inclusive.**

- Strongly agree
- Agree
- Neither agree nor disagree
- Disagree
- Strongly disagree

**11. Please list up to three proposals how diversity and inclusion can be promoted in science and clinical trials.**

**WORKING IN TRANSPLANTATION**

**12. In my workplace, clinical teams and research groups are inclusive and diverse across all levels of seniority.**

- Strongly agree
- Agree
- Neither agree nor disagree
- Disagree
- Strongly disagree

**13. Please list up to three proposals how to promote diversity and inclusion in clinical teams and research groups.**

**14. There are equal opportunities for training in transplantation and related disciplines.**

- Strongly agree
- Agree
- Neither agree nor disagree
- Disagree
- Strongly disagree

**15. Please list up to three proposals how equal opportunities can be granted in the context of training in transplantation and related fields.**

**16. There is equal access to publishing in transplantation and related fields.**

- Strongly agree
- Agree
- Neither agree nor disagree
- Disagree
- Strongly disagree

**17. Please list up to three proposals how publishing in transplantation and related fields can be made more equally accessible.**

**18. There are equal chances of career progression in transplantation and related disciplines.**

- Strongly agree
- Agree
- Neither agree nor disagree
- Disagree
- Strongly disagree

**19. Please list up to three proposals how equal chances for career progressions can be granted in transplantation and related fields.**

**EQUALITY IN HEALTHCARE AND TRANSPLANTATION**

**20. I believe that transplantation is equally accessible to all, regardless of gender, sexual orientation, ethnic background, socio-economic status, etc.**

- Strongly agree
- Agree
- Neither agree nor disagree
- Disagree
- Strongly disagree

**21. Please list up to three proposals how transplantation could be made accessible in a more equitable manner, regardless of gender, sexual orientation, ethnic background, socio-economic status, etc.**

**22. I believe that post-transplant care is equally accessible to all, regardless of gender, sexual orientation, ethnic background, socio-economic status, etc.**

- Strongly agree
- Agree
- Neither agree nor disagree
- Disagree
- Strongly disagree

**23. Please list up to three proposals how post-transplant care could be made accessible in a more equitable manner, regardless of gender, sexual orientation, ethnic background, socio-economic status, etc.**

**FINAL COMMENTS**

**24. Please share any additional thoughts and suggestions you may have about any aspect diversity and inclusion.**
